# Incidental Finding of Bilateral Acute Pulmonary Emboli in a Patient With Asymptomatic COVID-19 Infection

**DOI:** 10.7759/cureus.18496

**Published:** 2021-10-05

**Authors:** Htun M Aung, Aye M Thida, Melissa Fils-Aime, Malar Thwin

**Affiliations:** 1 Department of Medicine, Interfaith Medical Center, Brooklyn, USA; 2 Department of Medicine, Woodhull Medical and Mental Health Center, Brooklyn, USA

**Keywords:** elevated d-dimer, venous thromboembolism, anticoagulation, covid-19 infection, pulmonary emboli

## Abstract

A 58-year-old male with a history of hypertension, dyslipidemia, osteoarthritis of both knees, and morbid obesity presented to the emergency department for opioid detoxification. He complained of generalized soreness, anxiety, and difficulty sleeping but denied signs and symptoms suggestive of coronavirus disease 2019 (COVID-19) infection. His COVID-19 polymerase chain reaction (PCR) result came back positive, and his D-dimer level was 5373 ng/mL. A computed tomography pulmonary angiogram showed a moderate burden of bilateral acute pulmonary emboli. He was managed with enoxaparin sodium subcutaneous therapeutic dose for three days, followed by oral apixaban 10 mg twice a day for seven days and then 5 mg twice a day for six months. To date, the rate of venous thromboembolism (VTE) in nonhospitalized patients with COVID-19 has not been reported, and current guidelines do not recommend thromboprophylaxis for these patients.

## Introduction

Coronavirus disease 2019 (COVID-19) is a new disease caused by severe acute respiratory syndrome coronavirus 2 (SARS-CoV-2) [1]. Starting as a cluster of pneumonia cases in Wuhan, a city in the Hubei Province of China, it rapidly spread, causing an epidemic in China. It was followed by an increasing number of cases in other countries, becoming a global pandemic. According to the World Health Organization, there were over 229 million cases of COVID-19 infections, with over 4.7 million confirmed deaths worldwide as of 22 September 2021 [2].

Our understanding of the mysterious COVID-19 is still evolving. Studies have reported that COVID-19 was associated with increases in coagulation markers such as fibrin, fibrin degradation products, fibrinogen, and D-dimers, as well as inflammation, leading to a prothrombotic state [3,4]. In fact, a meta-analysis of 86 studies in hospitalized patients with COVID-19 found an overall venous thromboembolism (VTE) prevalence of 14.1% (95% confidence interval [CI]: 11.6-16.9) with 22.7% (95% CI: 18.1-27.6) in intensive care unit (ICU) patients and 7.9% (95% CI: 5.1-11.2) in non-ICU patients [5].

We present a case of an incidental finding of pulmonary embolism in a patient with asymptomatic COVID-19.

## Case presentation

A 58-year-old male with a history of hypertension, dyslipidemia, osteoarthritis of both knees, and morbid obesity presented to the emergency department for opioid detoxification. His triage vitals were as follows: body temperature of 98.8°F, blood pressure of 119/72 mmHg, heart rate of 70 beats per minute, respiratory rate of 18 breaths per minute, and oxygen saturation of 98% in room air. He complained of generalized soreness, anxiety, and difficulty sleeping but denied signs and symptoms suggestive of COVID-19, such as fever, chills, cough, shortness of breath, difficulty breathing, fatigue, headache, loss of taste or smell, sore throat, runny nose, nausea, vomiting, or diarrhea. A review of system was negative otherwise. He mentioned that he used two bags of heroin daily, two bags of cocaine every other day, and two 16 ounces of beer daily. He stated that he was enrolled in a methadone maintenance treatment program. Physical examination revealed a well-nourished man (body mass index of 39.6 kg/m^2^) with no acute respiratory distress. He had a Clinical Institute Withdrawal Assessment for Alcohol - Revised (CIWA-Ar) score of 4, but there were no signs of opioid or alcohol withdrawal.

Investigations

Most initial laboratory investigations were within normal limits, except for a positive COVID-19 polymerase chain reaction (PCR) test (Table 1). An electrocardiogram showed normal sinus rhythm, ventricular rate of 80 beats per minute, and corrected QT interval of 447 milliseconds. A chest X-ray did not reveal any acute process. Urine toxicology was positive for opioids and cocaine. Given a positive COVID-19 PCR result, he was admitted to the medical floor.

**Table 1:** Initial laboratory investigations

Investigations	Results	Normal range
White blood cells	6.0 x 10^3^/µL	4.5–11 x 10^3^/µL
Hemoglobin	13.4 g/dL	13–17 g/dL
Hematocrit	41.8%	39%–53%
Platelet count	256 x 10^3^/µL	130–400 x 10^3^/µL
Sodium	142 mmol/L	136–145 mmol/L
Potassium	3.5 mmol/L	3.5–5.1 mmol/L
Chloride	102 mmol/L	98–107 mmol/L
Carbon dioxide	29 mEq/L	23–31 mEq/L
Anion gap	11 mEq/L	6–12 mEq/L
Blood urea nitrogen	10.4 mg/dL	8.4–25.7 mg/dL
Creatinine	0.92 mg/dL	0.72–1.25 mg/dL
Glucose	95 mg/dL	80–115 mg/dL
Calcium	9.2 mg/dL	8.8–10.0 mg/dL
Magnesium	2.1 mg/dL	1.6–2.6 mg/dL
Phosphorus	3.1 mg/dL	2.3–4.7 mg/dL
Total bilirubin	0.4 mg/dL	0.2–1.2 mg/dL
Alkaline phosphatase	<10 U/L	10–55 U/L
Aspartate phosphatase	14 U/L	5–34 U/L
Coronavirus disease 2019 polymerase chain reaction test	Positive	Negative

**Figure 1:**
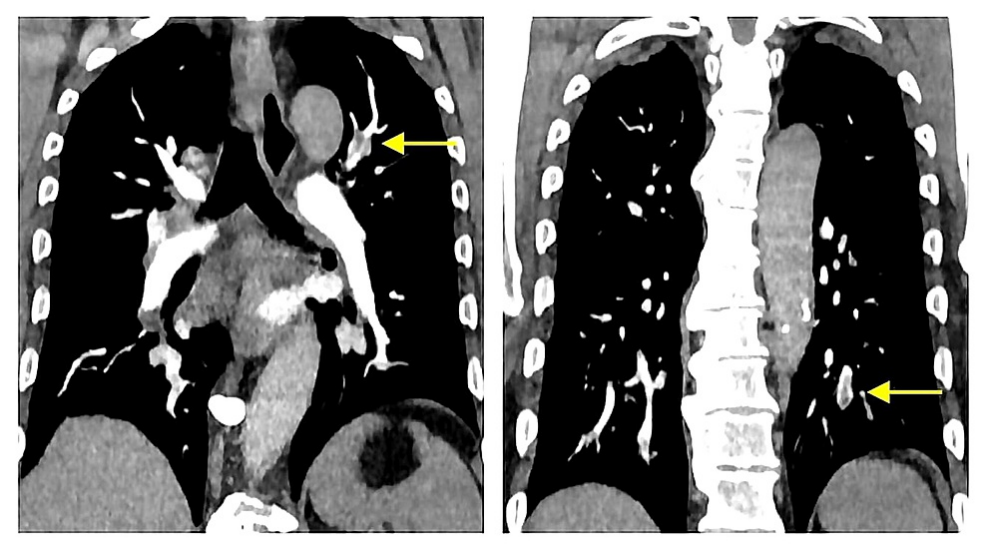
The computed tomography pulmonary angiogram showed a moderate burden of acute pulmonary emboli in branches to the left upper lobe, left lower lobe, and right lower lobe, and an embolus in the distal left main pulmonary artery

On further workup, his D-dimer level was elevated at 5373 ng/mL (normal range: 45-500 ng/mL). As his creatinine level was within normal limit, a computed tomography pulmonary angiogram was done, which showed a moderate burden of acute pulmonary emboli in branches to the left upper lobe, left lower lobe, and right lower lobe, and an embolus in the distal left main pulmonary artery (Figure 1).

Treatment

A diagnosis of pulmonary embolism was made. An arterial blood gas study was performed, which came back as normal. He was started on enoxaparin sodium subcutaneous therapeutic dose (1 mg/kg every 12 hours). As he developed signs and symptoms of alcohol withdrawal, he was placed on front-loading therapy with chlordiazepoxide and thiamine, folic acid, and multivitamin tablets as daily scheduled doses. He also received methadone maintenance treatment for opioid withdrawal.

After receiving enoxaparin sodium subcutaneous therapeutic dose for three days, he was started on apixaban 10 mg per oral (PO) twice a day for seven days according to the pulmonary and hematology teams.

Outcome and follow-up

The patient remained clinically asymptomatic throughout the hospital stay for four days and was discharged with home quarantine on day 5. He was recommended to finish the seven-day course of apixaban 10 mg PO twice a day and then to continue apixaban 5 mg PO twice a day for six months.

## Discussion

According to the National Institutes of Health (NIH), “although abnormalities in these coagulation markers have been associated with worse outcomes, prospective data demonstrating that the markers can be used to predict the risk of VTE in those who are asymptomatic or who have mild SARS-CoV-2 infection is lacking. In hospitalized patients with COVID-19, hematologic and coagulation parameters are commonly measured; however, there are currently insufficient data to recommend either for or against using such data to guide management decisions” [6]. In addition, “there are currently insufficient data to recommend either for or against routine deep vein thrombosis screening in COVID-19 patients without signs or symptoms of VTE, regardless of the status of their coagulation markers,” as stated by the NIH.

The risk of VTE in patients with COVID-19 following hospital discharge appears to be low and similar to other patients following a medical admission [7]. A placebo-controlled randomized controlled trial is being conducted to evaluate the benefit of thromboprophylaxis in these patients [8].

A literature review in the MEDLINE database using PubMed revealed several case reports and case series describing pulmonary thromboembolism in asymptomatic patients with COVID-19 [9-14]. To date, the rate of VTE in nonhospitalized patients with COVID-19 has not been reported. Besides, prospective data demonstrating that the coagulation markers can be used to predict the risk of VTE in patients with COVID-19 who are asymptomatic or who have a mild infection is not yet available. Current guidelines do not recommend thromboprophylaxis for nonhospitalized patients with COVID-19 [6].

## Conclusions

Our patient was found to have elevated D-dimer and a positive COVID-19 PCR result as a part of the admission investigations, leading to the incidental finding of acute pulmonary embolism on computed tomography pulmonary angiogram. To date, the rate of VTE in nonhospitalized patients with COVID-19 has not been reported, and current guidelines do not recommend thromboprophylaxis for nonhospitalized patients with COVID-19.
